# A Case Report of Spontaneous Vaginal Delivery Under General Anesthesia in a Patient With a Large Cerebral Aneurysm

**DOI:** 10.7759/cureus.53822

**Published:** 2024-02-08

**Authors:** Gregory W Kirschen, Lucy Brown, Joy Davis, Dan Kim, David J Berman, Timour Al-Khindi, Justin Caplan, Shannon M Osborne

**Affiliations:** 1 Gynecology and Obstetrics, Johns Hopkins University School of Medicine, Baltimore, USA; 2 Anesthesiology, Johns Hopkins University School of Medicine, Baltimore, USA; 3 Neurosurgery, Johns Hopkins University School of Medicine, Baltimore, USA

**Keywords:** subarachnoid hemorrhage, cerebral perfusion pressure, icp: intracranial pressure, valsalva maneuver, second stage of labor

## Abstract

Cerebral aneurysms are rarely encountered in pregnancy. Their antepartum and intrapartum management remain clinically challenging, primarily due to concern regarding potential rupture. We present a case of a patient in preterm labor at risk for imminent delivery with a 10mm cerebral aneurysm. She was recommended for cesarean section (CS), yet delivered via spontaneous vaginal delivery in the operating room after induction of general anesthesia for the intended CS. Her aneurysm and neurologic function remained intact postpartum. Cerebral aneurysms <5mm are unlikely to undergo significant growth during pregnancy. The presence of a cerebral aneurysm is not automatically a contraindication to the Valsalva maneuver. The recommendation for which patients with unruptured cerebral aneurysms should deliver by CS, operative vaginal delivery, or unassisted vaginal delivery (i.e., which patients should avoid Valsalva maneuver intrapartum), is complex and requires multidisciplinary discussion.

## Introduction

Pregnancy can increase the likelihood of an intracranial hemorrhage up to five-fold, and subarachnoid hemorrhage (SAH) from aneurysms may account for nearly 1 in 10 of all maternal deaths [1]. Increased levels of estrogen, progesterone, human chorionic gonadotropin, and relaxin mediate changes in the arterial and venous intima and media that weaken the vessel walls, thereby predisposing pregnant patients to formation, enlargement, and rupture of aneurysms [2]. Moreover, the pregnancy-related drop in systemic venous resistance (SVR) and increase in cardiac output cause shear stress on already structurally weak vessels [3].

Among non-pregnant patients, management of small (<7mm) and medium to large-sized (7-12mm) aneurysms is controversial, depending largely on comorbidities and risk factors, such as smoking [2]. The risk of rupture for small to medium-sized aneurysms in the general population is estimated to be 0-2.6% per year; therefore, the International Study of Unruptured Aneurysms proposed that small to medium-sized aneurysms without a history of SAH can be managed conservatively [1]. Unruptured aneurysms with associated headaches, cranial nerve palsies, lobulations, or evidence of serial growth are considered high risk and may necessitate intervention [1]. Some data suggest that newly formed, small aneurysms also have high rates of rupture [4]. It is uncertain whether given the physiologic changes and vascular stress of pregnancy, a more aggressive approach to small, asymptomatic aneurysms is warranted.

While historically cesarean section (CS) has been preferred in patients with unruptured aneurysms due to risks of high-pressure Valsalva, there are no evidence-based recommendations for delivery management [2]. Notably, aneurysm rupture has been found in a small case series of weight trainers, lending credence to the idea that Valsalva may increase rupture risk although the absolute risk remains unknown [5]. The risk of aneurysmal SAH has been shown to be similar in pregnant and laboring patients compared to the general population, although whether this holds true when stratifying by aneurysm size is yet to be determined [6]. Effective neuraxial analgesia alongside operative delivery methods may reduce the risk of prolonged Valsalva and expulsive efforts [6]. CS has not been shown to have superior maternal and fetal outcomes compared to vaginal delivery [2]. However, severe circumstances where CS would be indicated include maternal coma, brainstem damage, and aneurysm diagnosis at the time of labor [7]. Decisions regarding the mode of delivery should be based on obstetric considerations and shared decision-making.

Here, we present a case of a patient with a 10mm cerebral aneurysm who was taken for an emergent primary CS and delivered vaginally under general anesthesia (GA). The patient’s written informed consent was obtained.

## Case presentation

A 24-year-old gravida 3 para 2 woman at 35 weeks gestation presented to labor and delivery with painful contractions every 2-3 minutes. Fetal heart tracing was category I. She had a spontaneous amniotomy and her cervix was found to be 5cm dilated.

**Figure 1:**
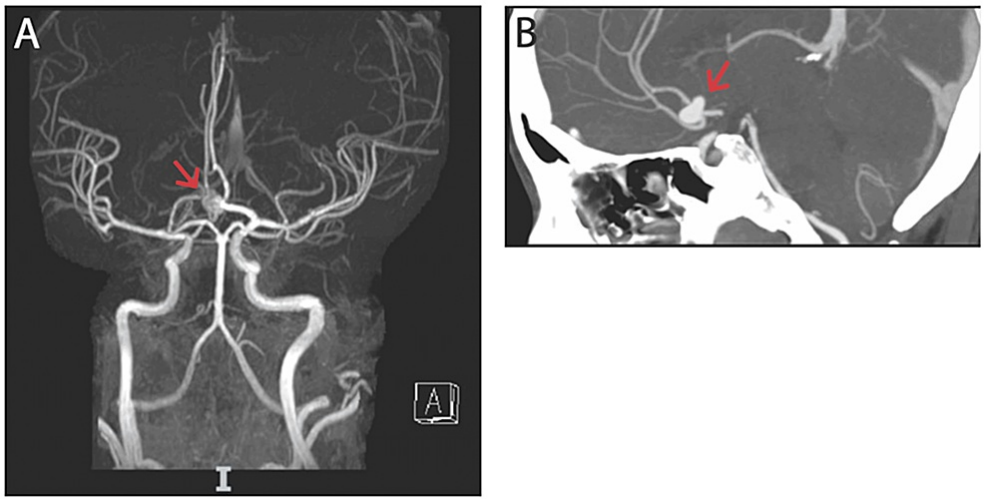
Imaging of patient’s cerebral vasculature A) Magnetic resonance angiogram of the head (coronal view) with intravenous contrast demonstrating stable 10mm saccular aneurysm (arrow) at the junction of the anterior communicating artery and the left A1 segment. The right A1 segment is hypoplastic. B) Computed tomography angiogram of the head demonstrating the same aneurysm in the midsagittal section (arrow).

A review of the electronic medical record revealed one emergency department visit six months prior for a complaint of headache and blurry vision. During that visit, she was discovered to be pregnant. Computerized tomography angiography was performed which demonstrated a large anterior communicating artery aneurysm. Magnetic resonance imaging of the brain further characterized a 10mm saccular aneurysm arising from the left anterior communicating artery without evidence of SAH (Figure 1).

A lumbar puncture was performed given her headache and identified aneurysm, which ruled out SAH. The patient was a non-smoker, without a family history of cerebral aneurysms. Neurosurgery was consulted and the patient was ultimately discharged with a plan for outpatient high-risk obstetrics and neurosurgery follow-up. Unfortunately, the patient was lost to follow-up (having multiple barriers to care including language barrier, uninsured status, lack of social support, and poor health literacy) until presentation in preterm labor.

Upon arrival to labor and delivery, anesthesiology and neurosurgery were consulted. Neurosurgery recommended maintaining systolic blood pressure (SBP) <140mmHg in the peripartum setting to minimize the risk of aneurysm rupture. Within 15 minutes, she progressed to full dilation with fetal head at zero station. She did not have neuraxial anesthesia and her blood pressures were noted to be 130-140/60-80mmHg. No provider was immediately available to provide forceps-assisted vaginal delivery. Due to concern for potential aneurysm rupture, a decision was made to perform an emergent CS under GA.

In the operating room, GA was induced with propofol and succinylcholine, and the patient’s trachea was intubated easily. Immediately upon induction and prior to skin incision, the fetal head was palpated to be descending in the pelvis. The sterile drapes were lifted, revealing a fetal head delivered in the right occiput anterior position. The remainder of the body and placenta were delivered vaginally. GA was reversed and the patient was transferred to the recovery unit where she required several doses of intravenous beta blockers to maintain SBP <140mmHg.

Close neurologic and hemodynamic monitoring were provided in an intensive care unit overnight. She was downgraded to the postpartum floor on postoperative day one and recovered as expected following vaginal delivery. She remained inpatient for several days for neurosurgical planning and care coordination. A diagnostic cerebral angiogram performed on postpartum day six showed no change in aneurysm size compared to before delivery. She was ultimately discharged on postpartum day nine with plans for craniotomy and aneurysm clipping at six to eight weeks postpartum.

## Discussion

To understand how pregnancy influences cerebral aneurysms, it is first important to consider hemodynamics and intracranial circulation both inside and outside of pregnancy. Maternal total blood volume increases by ~40% by the third trimester, and cerebral blood flow volume through the internal carotid artery increases by 16% from the first to the third trimester [8,9]. Aneurysms are sensitive to both blood volume and pressure in the cerebral circulation, which is relevant, especially with the Valsalva maneuver during the second stage of labor. A transcranial Doppler study of the middle cerebral artery in non-pregnant healthy adult volunteers demonstrated that performing the Valsalva maneuver for 15 seconds led to an increase in arterial blood pressure of 30%, accompanied by an initial drop in cerebral blood flow velocity (CBFV) followed by an “overshoot” above a baseline of 56% [10].

The risk of aneurysm rupture is proportional to a transmural pressure gradient (i.e., the difference in pressure within the aneurysm, approximated by mean arterial pressure (MAP), and outside the aneurysm, approximated by intracranial pressure (ICP)). A proxy for transmural pressure gradient is cerebral perfusion pressure (CPP), defined as MAP - ICP. Several studies indicate that the Valsalva maneuver decreases CPP [11,12], through an increase in ICP [13,14]. CPP is a proxy for transmural pressure, thus high CPP is associated with risk of aneurysm rupture. In the ruptured aneurysm setting, there is a concern that, for instance, placing an external ventricular drain will lower the ICP too greatly, increasing the transmural pressure gradient and leading to aneurysm rupture. High ICP on the other hand should be protective in Valsalva and the pregnant setting.

In pregnant subjects, maternal CBFV in 22 laboring patients was measured via transcranial Doppler, 7 with epidural anesthesia and 22 without [15]. In unepiduralized patients, estimated CPP (eCPP) dropped while cerebrovascular resistance rose significantly at the peak of contractions and during pushing, while cerebral blood flow did not change. In epiduralized patients, contractions and pushing did not affect eCPP, cerebrovascular resistance, or cerebral blood flow. Corroborating these findings, a study of 26 healthy women in labor with epidural anesthesia measured intraocular pressure and mean ocular perfusion pressure and found no significant differences in these metrics during the various stages of labor [16]. Given these findings, we can start to understand the potential hazards of increased blood volume, increased cerebral circulation, and changes in intracranial vascular resistance and pressure during labor.

The overall risk of cerebral aneurysm rupture in pregnancy is approximately 1.4%, compared with the annual risk of 1.9% in non-pregnant all-comers [17,18]. Outside of pregnancy, the risk is higher in women, symptomatic individuals, aneurysm size > 10mm, or aneurysms in the posterior cerebral circulation [18]. Based on a case series, small aneurysms (≤5mm) are unlikely to change in size during pregnancy [19]. Considering all women with cerebral aneurysms in pregnancy, the risk of rupture is greatest during the third trimester and when size >10mm [2].

It is unclear which patients with cerebral aneurysms should be counseled to avoid Valsalva and deliver by cesarean delivery or via assisted second stage of labor (i.e., vacuum or forceps). While no formal guidelines on recommended modes of delivery exist, an interdisciplinary discussion between providers within obstetrics/maternal-fetal medicine, obstetric anesthesiology, and neurosurgery is advisable. Approximately 70% of pregnant women with known cerebral aneurysms undergo planned CS based on a large retrospective database [17]. Despite the high CS rate among those with aneurysms, there is no evidence recommending against a trial of labor in these patients, especially with smaller (<10mm) aneurysms. Rather, clinicians rely on data extrapolated from non-pregnant individuals demonstrating that activities related to Valsalva such as startling, sexual intercourse, and strenuous physical exertion, are associated with a sudden change in pressure across the aneurysm wall, may precipitate rupture and SAH [20].

## Conclusions

In summary, we report a patient with a 10mm cerebral aneurysm for which the decision was made to avoid Valsalva, who unexpectedly delivered vaginally without Valsalva under GA before skin incision during attempted emergency CS. The case highlights management challenges and ambiguity in cases of aneurysm in pregnancy which warrants multidisciplinary discussion and collaboration to optimize maternal and neonatal outcomes.
